# Comparison of the planimetry and point-counting methods for the assessment of the size of the mandible cysts on orthopantomograms

**DOI:** 10.4317/medoral.17570

**Published:** 2011-12-06

**Authors:** Emel Bulut, Bünyamin Şahin, Mehtap Muğlalı, Burak Bekçioğlu

**Affiliations:** 1 Associate Professor, PhD, DDS, Ondokuz Mayis University, Faculty of Dentistry, Department of Oral and Maxillofacial Surgery, Samsun, Turkey; 2 Professor, PhD, Ondokuz Mayis University, Faculty of Medicine, Department of Anatomy, Samsun, Turkey; 3 Associate Professor, PhD, DDS, Ondokuz Mayis University, Faculty of Dentistry, Department of Oral and Maxillofacial Surgery, Samsun, Turkey; 4 Assistant Professor, PhD, DDS, Ordu University, Faculty of Dentistry, Department of Oral and Maxillofacial Surgery, Ordu, Turkey

## Abstract

Objective: The purpose of this study is to compare the computer-assisted planimetry and point-counting methods in evaluating the sizes of the mandibular cysts with respect to their agreement and the time required to analyze. Study Design: The surface areas of 46 mandibular cyst lesions on orthopantomograms were estimated using the point-counting and computer-assisted planimetry methods. Three observers evaluated the outlined areas twice, using the point-counting (PC) and computer-assisted planimetry (CAP) methods with an interval of two weeks. In the planimetry technique, digitalized images and ImageJ software were used to measure the surface area of the half mandibles and cysts. The grids were superimposed over the same images and the number of points hitting the interested structures was counted for the point-counting technique. The projection area fraction (PAF) of the cysts within the mandible was estimated using the obtained values by means of the two techniques. Intraclass correlation coefficient was used to assess the level of agreement between the two methods. Inter-rater reliability analysis using the Kappa statistic was performed to determine consistency among raters. Results: CAP and PC techniques showed consistent intra-observer values in all observers. Intraclass correlation between CAP and PC measurements of first, second and third observers were found to be 0.9986, 0.9988 and 0.9994 respectively. The durations of PC technique was 32% higher than the CAP technique. Conclusion: PC and CAP methods were seemed to show sufficient agreement to be used interchangeably. The main disadvantage of the PC analysis is it takes more time than CAP method.

** Key words:**Orthopantomogram, mandible, cyst, point-counting, planimetry.

## Introduction

The ability to objectively measure surface areas of cystic lesions on radiography is important especially in evaluation of the degree of the treatment respons. Several methods have been described for assessing the surface area of maxillofacial lesions and swellings on Computerized Tomography (CT) scans or Magnetic Resonance (MR) images and stereophotogrammetry; those that give three dimensional measurements are the two that are most advantageous (1-3).

However, these methods have some disadvantages such as high cost, claustrophobic properties, the need for complex machinery, loss of time and etc. Therefore, they are not used routinely (4).

Roentgenograms show the two-dimensional reflection area of three-dimensional objects. Thus roentgenograms are projections of structures or organs exposed to X-ray radiation. The projection areas of these two-dimensional images also can be used to assess the magnitude of the structures examined for research or clinical purposes (5).

 Similarly, an orthopantomogram (OPG) is the chief imaging procedure used to monitor cysts. It is a low cost and quick method for evaluating cysts; however, it provides no quantitative information about the size of the lesions. Although some automatic machines or their software can measure the contour of an object to obtain the projection areas, this is a simple method to evaluate the size of pathological or normal subjects or organs. However, if the images do not include a scale, the size differences in certain subjects cannot be monitorized with the aid of the direct roentgenograms (4,6).

The aim of this study was comparing the computer-assisted planimetry (CAP) and (PC) methods in evaluating the sizes of the mandible cysts. The fractions between the cysts and half mandibles were used to standardize the evaluation of the cyst sizes.

## Material and Methods

Eighty half mandibles from 40 patients were evaluated in this study. Cysts at the midline region extending to the both halves of mandibles were evaluated as two separated cyst regions, and a total of 46 cystic areas in orthopantomograms were evaluated. CAP method was considered as “gold standard” and PC technique was compared with this technique.

-Point-counting technique

**Figure 1:**
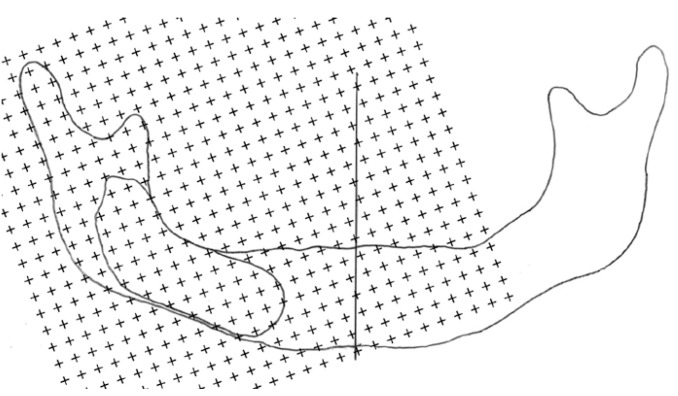
A hand drawing of the boundaries of the mandible and cyst under the guidance of the orthopantomogram. A PC grid with 6 mm between the plusses was superimposed over the drawing to assess the projection area of the mandible.

Transparent papers were placed on the radiographs and the boundaries of the mandibles (46 samples) and cysts (21 on the right side and 25 on the left side) were delineated with a permanent pen. The midlines of the mandibles were marked by a straight line in the centre. To estimate the number of points, transparent sheets with points (+) on it was randomly superimposed on lesions and half mandible projection areas. The distances between the constant angles of crosses were 2 millimeters for cystic areas and 6 mm for half mandibles. The numbers of intersections (i.e. upper right corner of the crosses) hitting the area of interest were counted (Fig. 1) The projection area fraction (PAF) of the cysts within the half mandible was estimated using the following formula:

ΣPcyst indicates the number of points hitting the cyst and ΣPhalfmandible is the number of points hitting the half mandible. The unit of the final data is a percentage.

Three independent observers evaluated the outlined areas twice using the PC method with an interval of two weeks.

-Planimetry technique

**Figure 2:**
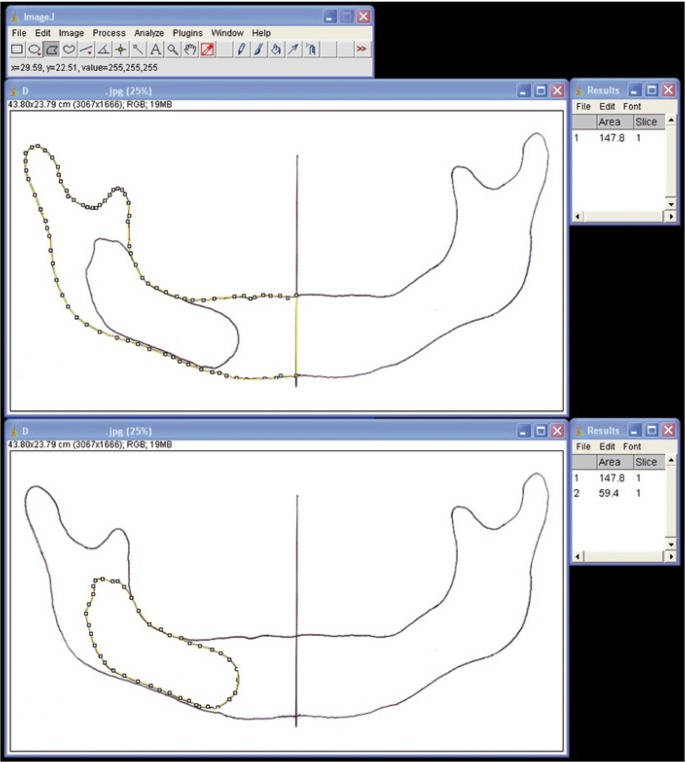
Application of the CAP technique for the assessment of the projection areas of the half mandible (above) and cyst (below) using the ImageJ software

A straight line with a known length was drawn on the hand drawings of the orthopantomograms. The hand drawings were scanned and stored in “jpeg” format with true colour class and 3510×2550 pixel resolution. These digital images were used to measure the surface area of the half mandibles and cysts using ImageJ software, which is a freeware distributed by the National Institute of Health (USA). For this purpose, the images were transferred to the ImageJ software. The straight line with a known length on the films was used to set the scale of the program. The polygon selection tool was used to delineate the outermost boundaries of the mandibles and cysts (Fig. 2). The program automatically measured the delineated area of the images. The obtained area values were used to estimate the PAF of the cysts within the half mandible using the following formula:

Acyst indicates the surface area of the cyst and Ahalfmandible is the surface area of the half mandible. The unit of the final data is a percentage.

These procedures were repeated on films two weeks later using the same estimation parameters by the same three observers.

The processing times for both PC and CAP techniques were recorded. Intraclass correlation coefficient values were determined to assess the level of agreement between the two methods for each observer. An inter-rater reliability analysis using the Kappa statistic was performed to determine consistency among observers.

## Results

The mean PAF values (±SEM) in point-counting technique for first and second session were 11.9±0.9% and 12.0±0.9%, respectively. In planimetry technique, the mean PAF values were 11.6±0.9% and 11.7±0.9% for the first and sessions, respectively. The mean PAF values evaluated with PC and CAP techniques for two sessions by three observers are shown in ( Table 1).

**Table 1:**
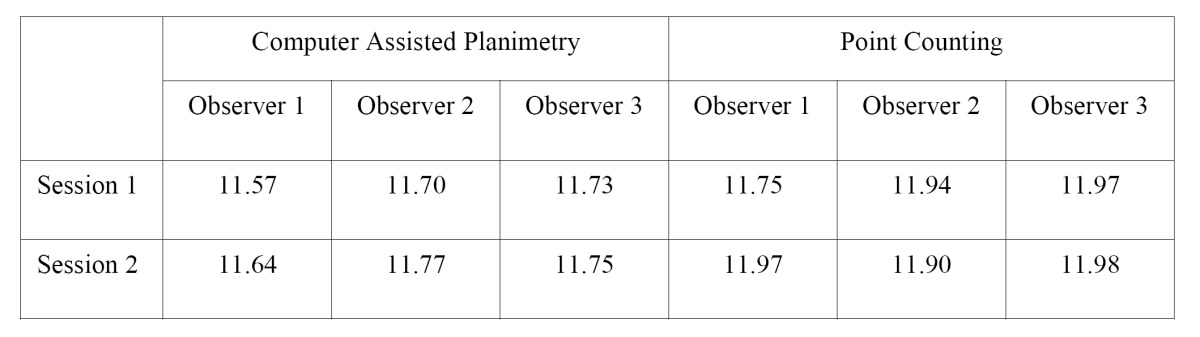
Estimated average PAF values (%) using PC and CAP methods.

Intraclass correlation between CAP and PC measurements of first, second and third observers were found to be 0.9986 (95% confidence interval: 0.9976-0.9992), 0.9988 (95% confidence interval: 0.9981-0.9993) and 0.9994 (95% confidence interval: 0.9989-0.9997), respectively.

Inter-observer agreement was found to be almost perfect in both techniques for all observers. Weighted kappa and standard error values for each observers and sessions are shown in ( Table 2).

**Table 2:**
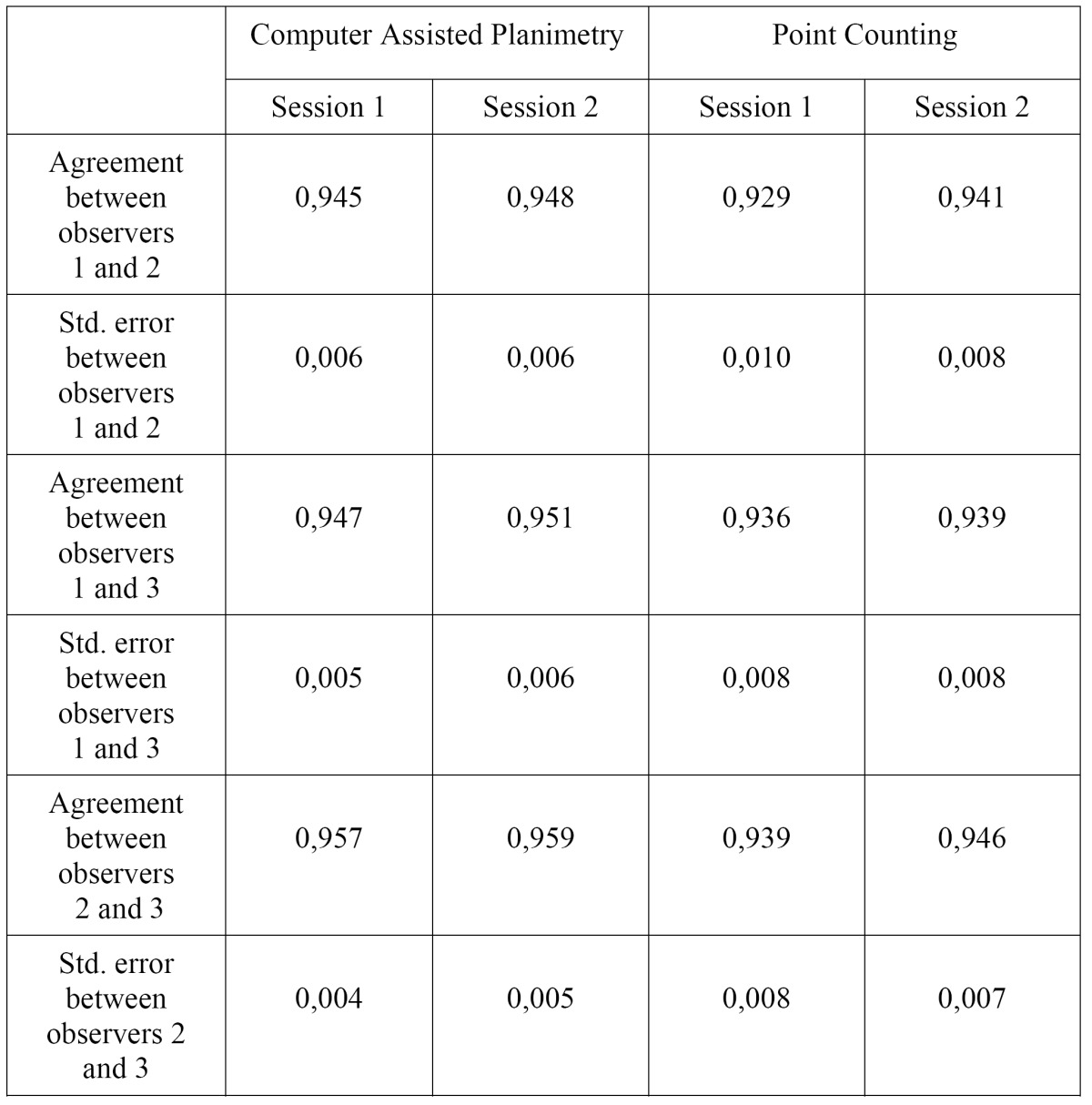
Agreement (weighted Kappa) and standard error values for each observers and sessions.

**Figure 3:**
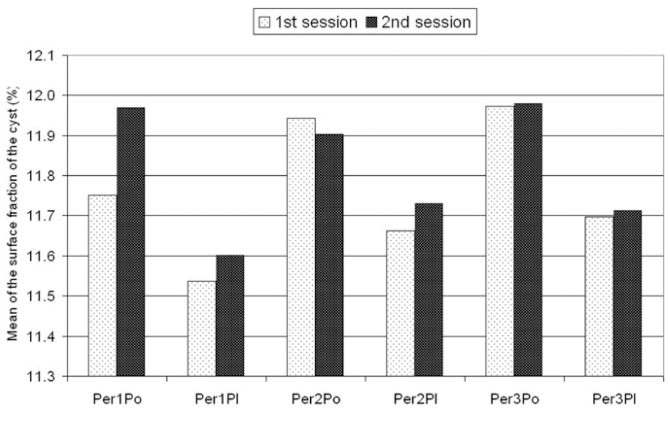
A graphic showing the comparison of point-counting and planimetry techniques. Per; person, Pl; planimetry, Po; point-counting.

Inter-observer, intra-observer values and the comparison of PC and CAP techniques are shown in the graphic (Fig. 3).

The durations of PC and CAP approaches were evaluated. The mean duration is determined as 03:22±01:07 (Minimum 01:22, Maximum 7:56) minutes for PC procedure and 02:33± 01:13 (Minimum 00:57, Maximum 06:40) minutes for CAP procedure. The CAP took less time (32%) than the point-counting technique.

## Discussion

Digital photography with CAP is a reliable and accurate method and provides a digital image recording. This process is known as image digitalization. Each pixel in the image is individually sampled and its brightness measured and quantified. This integer value is stored in the corresponding pixel of the computers bitmap image. The area that defines the object’s outline is reported as a polygon area and in order to measure this, certain computer programs using this digitization technique can be employed. The ImageJ analyzing program is one of the most commonly used program in practice.

PC is a simple accurate and practical technique, which is widely used in the estimation of the irregularly shaped sectional surface area to obtain the volume of organs and structures using macroscopic, microscopic or radiological images (4,6). Although this is not a new method, the use of a PC technique to measure the cysts area drawn on orthopantomogram is not described in the literature. In this study, CAP is considered as “gold standard” and PC technique is compared with this technique to find out if they can be used interchangeably.

The PC grid, which has some point sets at distinct densities on a transparent sheet, can be used to estimate the surface area (4). Minimal variations in measuring between different observers and inability of performing a volumetric measurement are disadvantages of this method.

Evaluation of the dimensions of bone lesions is very important especially in observing the prognosis of the lesions or determining the degree of treatment in healing period. In oral and maxillofacial surgery, orthopantomograms are often used to diagnose the lesions and to follow the differences from time to time. Radiographs are two-dimensional images and surface area at the orthopantomogram is a commonly used tool to determine the size of the lesions (7). Therefore, measuring and evaluating the surface area must be standardized to follow the prognosis of the bone lesion. CAP is the most commonly used approach, which provides the surface area values of the projections for organ volume determinations (8-11). It can also be used for surface area measurements (12) and we used PC technique as an alternative. A comparative study of CAP and PC methods was reported by Aydin et al. (13) in evaluating surface areas of dermatologic lesions. In our study, two techniques are compared with regard to the agreement and required time. This study was also designed to evaluate inter- and intra-observer reliability and accuracy of PC method for mandibular bone lesion surface area measurement. To minimize the inter-observer variations depending on the delineation of boundaries of the interested structures, we prefer to analyze the drawings with PC and CAP.

Our results showed that there was a high agreement in intra-observer results both in the PC and CAP techniques. Therefore, these two techniques can be considered as reliable techniques with low intra-observer variation. However, there are some drawbacks of the PC technique. For instance, variations can be occurred originating from the thickness of the permanent pen, which is used in transferring the lesion and mandible margins from OPG to transparent sheet. Observer may judge the superimposition of the intersections of the plusses over the thickness of the pen. This may cause the exclusion or inclusion of the area of the line drawn by pen. For example, one observer may count the middle point of the plus which is on the line and the other may not. In addition, in CAP technique, one observer can use the external border of the line and the other may use the internal border. The differences can be due to these factors. The same procedure may cause the difference between the CAP and PC. However, the high inter-observer and intra-observer agreements make the technique reliable and repeatable. Additionally, the possibility of distortion was tried to be standardized by taking the orthopantomograms with the same X-ray device and at the same position for each patient.

We measured the half mandible areas with larger scaled grid (6 mm), but smaller scaled grids (2 mm) were used to measure the bone lesion areas. The application of the small grid increased accuracy and it didn’t affect the results because we evaluated the ratios between half mandibles and lesions in all measurements. Accuracy increased when the density of the grids decrease, however, it caused an increase in PC time.

CT scans or MR images and stereophotogrammetry; those that give three-dimensional measurements are the two that are most advantageous (3). However, these methods have some disadvantages such as high cost, claustrophobic properties, the need for complex machinery, and loss of time. Therefore, they are not practiced routinely (4). However, the presented method has the advantages of lower costs and radiation doses.

PC is an inexpensive and fast method, which can be applied without changing the routine procedure in daily practice. The time elapsed for CAP technique was found to be less than the PC technique, but scanning the orthopantomograms and the process which was needed to transfer the mandible views to the program were not evaluated, only the time of planimetry processes with the ImageJ software were measured. So, PC may still be supposed to be faster than the CAP method.

Our purpose was to compare two methods to assess the PAF between the surface areas of mandibular lesions and mandible. We evaluated the PAF because of the orthopantomograms can have different magnification degrees. The proposed method abolishes the effects of different magnification degrees to assess the size of the mandibular cysts.

The method described in this study represents a simple, practical, and inexpensive technique to evaluate the bone lesion surface area. As a result, both the PC technique and CAP method can be used for the evaluation of treatment or the follow-up process of mandibular lesions.
